# The Major Outer Membrane Protein P5 Binds Vitronectin to Mediate Serum Resistance in Nontypeable *Haemophilus influenzae*

**DOI:** 10.1093/infdis/jiaf489

**Published:** 2025-09-22

**Authors:** Sandra Jonsson, Martina Janoušková, Vaishnavi Venkatesh Rao, Junkal Garmendia, Yu-Ching Su, Kristian Riesbeck

**Affiliations:** Clinical Microbiology, Department of Translational Medicine, Faculty of Medicine, Lund University, Malmö, Sweden; Clinical Microbiology, Department of Translational Medicine, Faculty of Medicine, Lund University, Malmö, Sweden; Clinical Microbiology, Department of Translational Medicine, Faculty of Medicine, Lund University, Malmö, Sweden; Instituto de Agrobiotecnología, Consejo Superior de Investigaciones Científicas (IdAB-CSIC)-Gobierno de Navarra, Mutilva, Spain; Centro de Investigación Biomédica en Red de Enfermedades Respiratorias, Madrid, Spain; Clinical Microbiology, Department of Translational Medicine, Faculty of Medicine, Lund University, Malmö, Sweden; Clinical Microbiology, Department of Translational Medicine, Faculty of Medicine, Lund University, Malmö, Sweden

**Keywords:** complement inhibitor, nontypeable *Haemophilus influenzae*, OMP P5, serum resistance, vitronectin

## Abstract

Acquisition of complement regulators is a virulence strategy used by nontypeable *Haemophilus influenzae* (NTHi) to evade complement-mediated killing by the host. The major outer membrane protein of NTHi, P5, binds C4b-binding protein and factor H to promote bacterial serum resistance. We show that P5 also binds vitronectin, which inhibits the formation of the membrane attack complex at the terminal stage of the complement pathway. Heterologous surface expression of P5 variants from NTHi strains 3655, KR271, KR317, and P652 promoted vitronectin binding to the P5-expressing *Escherichia coli*. In contrast, deletion of P5 from the NTHi strains reduced vitronectin binding. Vitronectin acquisition conferred serum resistance to P5-expressing *E. coli*, but not to NTHi Δ*ompP5* mutants. Using site-directed mutagenesis, extracellular loop 2 of the P5 variants was identified as the binding site for vitronectin. In conclusion, our findings highlight P5 as a receptor for vitronectin that promotes NTHi serum resistance.

## Teaser

The major outer membrane protein P5 of nontypepable *Haemophilus influenzae* interacts with complement regulators including vitronectin. This is an immune evasion strategy used by bacteria for prevention and protection from the bactericidal activity of the human complement system.

Nontypeable *Haemophilus influenzae* (NTHi) is a gram-negative, human-specific bacterium commonly found as a commensal at the nasopharyngeal site [1]. NTHi acts as an opportunistic pathogen causing noninvasive diseases such as acute otitis media, conjunctivitis, and sinusitis, especially in children. NTHi is also associated with exacerbations in patients with chronic obstructive pulmonary disease (COPD) [2, 3]. Since the introduction of a vaccine against the encapsulated *H. influenzae* type b, the incidence of diseases caused by NTHi has significantly increased [4, 5].

The outer membrane protein (OMP) P5 of NTHi is part of the OmpA-like protein family. P5 of NTHi is composed of 2 domains: (1) a conserved N-terminal transmembrane domain with 4 nonconserved extracellular and hypervariable surface-exposed loops (loops 1 to 4); and (2) a conserved periplasmic C-terminal domain that interacts with peptidoglycan [6–9]. As a multifunctional virulence factor of NTHi, P5 plays an important role in bacterial evasion of the host immune defense and in host colonization. During infection, P5 functions as an adhesin that interacts with mucin, intracellular adhesion molecule 1 (ICAM-1), and carcinoembryonic antigen-related cell adhesion molecule 1 (CEACAM-1), promoting bacterial adherence to airway epithelial cells [10, 11]. P5 contributes to bacterial serum resistance by binding to C4b-binding protein (C4BP) and factor H (FH), thereby inhibiting the complement system [12–15].

Vitronectin is a glycoprotein, mainly produced in the liver and present in plasma at an average concentration of 200–400 µg/mL [16, 17]. Circulating vitronectin in blood is in monomer form with a molecular weight of 65–75 kDa. Vitronectin inhibits the formation of the membrane attack complex (MAC) by preventing the insertion of the C5b-7 complex into the cell membrane [18]. Vitronectin is also found in the airway mucosa, deriving from plasma exudation or locally produced by resident cells in lung tissue [19, 20]. Both vitronectin and FH can be detected at 0.3–20.6 nM and 2.1–17.2 nM, respectively, in human bronchoalveolar lavage fluid [21, 22]. The C4BP concentration has, however, not been fully defined.

Besides binding to C4BP and FH, NTHi also hijacks vitronectin to prevent the insertion of the MAC for serum survival [23–25]. We have previously shown that NTHi expresses several surface-associated virulence factors, that is protein E (PE), protein F (PF), and lipoprotein P4, which all bind vitronectin [26–30]. Here, we used a heterologous expression model, mutagenesis, and protein structure molecular analysis to identify P5 as an important vitronectin-binding protein of NTHi. Our finding reveals a novel function of this major OMP in vitronectin-associated serum resistance, adding to its previously described role in NTHi serum survival via C4BP and FH acquisition.

## METHODS

### Bacterial Strains and Culture Conditions

Bacterial strains used in the current study are listed in Table 1. NTHi wild-type and mutants were routinely cultured in brain-heart infusion liquid broth supplemented with β-nicotinamide adenine dinucleotide hydrate (Sigma-Aldrich) and hemin (Sigma) each at 10 µg/mL, or on chocolate agar. NTHi were cultured at 35.5°C in 95% humidity atmosphere containing 5% CO_2_. NTHi isogenic Δ*ompP5* mutants were cultured in the presence of 10 µg/mL chloramphenicol. *Escherichia coli* DH5α and BL21(DE3) were grown in Luria-Bertani (LB) liquid broth or on LB agar, whereas *E. coli* expressing P5 were cultured in the presence of 100 µg/mL ampicillin (Sigma).

**Table 1.** Bacterial Strains Used in This Study

Bacterial Strain	Description/Genotype^a^	References
NTHi 3655	Clinical isolate from a 10-year-old child with acute otitis media.	[31]
NTHi 3655Δ*ompP5*	Cm^R^. Isogenic mutant strain of NTHi 3655 with *ompP5* gene replaced with *cat* gene. The mutant strain is devoid of P5 expression.	[12]
KR271	Clinical isolate from blood culture of a 75-year-old individual with bacteremia.	[4, 26]
KR271Δ*ompP5*	Cm^R^. Isogenic mutant strain of KR271 with *ompP5* gene replaced with *cat* gene. The mutant strain is devoid of P5 expression.	[12]
KR317	Clinical isolate from an infant with otitis media.	[4, 26]
KR317Δ*ompP5*	Cm^R^. Isogenic mutant strain of KR271 with *ompP5* gene replaced with *cat* gene. The mutant strain is devoid of P5 expression.	This study
P652	Lower respiratory tract isolated from a patient with COPD.	[32]
P595	Lower respiratory tract isolated from a patient with COPD.	[32]
86-028NP	Clinical isolate from a child with chronic otitis media.	[33]
*E. coli* DH5α	Cloning host.	Invitrogen
*E. coli* BL21(DE3)	Protein expression host.	Invitrogen
*E. coli* DH5α-pET16b	Amp^R.^ *E. coli* DH5α bearing the empty vector pET16b.	This study
*E. coli* BL21(DE3)::*ompP5*^3655^	Amp^R^. *E. coli* BL21 (DE3) carrying recombinant plasmid of pET16b with *ompP5* ORF insertion expressing P5^3655^ at the surface.	[12]
*E. coli* BL21(DE3)::*ompP5*^KR271^	Amp^R^. *E. coli* BL21 (DE3) bearing recombinant plasmid of pET16b with *ompP5* ORF insertion expressing P5^KR271^ at the surface.	[12]
*E. coli* BL21(DE3)::*ompP5*^KR317^	Amp^R^. *E. coli* BL21 (DE3) bearing recombinant plasmid of pET16b with *ompP5* ORF insertion expressing P5^KR317^ at the surface.	This study
*E. coli* BL21(DE3)::*ompP5*^P652^	Amp^R^. *E. coli* BL21 (DE3) bearing recombinant plasmid of pET16b with *ompP5* ORF insertion expressing P5^P652^ at the surface.	This study

Abbreviations: Amp^R^, resistant to ampicillin; Cm^R^, resistant to chloramphenicol; COPD, chronic obstructive pulmonary disease; ORF, open reading frame.

^a^Concentrations of antibiotics used in the culture: 100 µg/mL ampicillin; 10 µg/mL chloramphenicol.

### Recombinant Protein Expression of P5 Variants in *E. coli*

The complete open reading frame of P5 was amplified from the genomic DNA of NTHi strains KR317 and P652 using primers with specific restriction sites (Supplementary Table 1). The resulting amplicon was digested and cloned into the expression vector pET16b(+) (Novagen) for protein expression. All recombinant plasmids (P5-pET16b) were maintained in *E. coli* DH5α.

Surface expression of P5 in *E. coli* was carried out as described [12]. *E. coli* BL21(DE3) transformed with P5-pET16b was induced with 1 mM isopropyl β-Ɒ-thiogalactoside (IPTG). Surface expression of P5 was detected by flow cytometry using rabbit anti-P5_Loop3^3655^ or anti-P5_Loop4^3655^ polyclonal antibodies (pAb) and a fluorescein isothiocyanate (FITC)-conjugated goat anti-rabbit pAb (Abcam) [12].

### Construction of *ompP5* Knockout Mutants in NTHi

The strategy to delete P5 (Δ*ompP5*) in NTHi KR317 and P652 was as previously described (Supplementary Figure 1) [12, 30]. The chloramphenicol acetyltransferase gene (*cat*) was used to replace *ompP5*. Bacteria were transformed with a DNA fragment consisting of *cat* inserted between the upstream and downstream flanking regions of the *ompP5* [34]. The yielded Δ*ompP5* mutants were selected by chloramphenicol (10 µg/mL; Sigma), and verified by polymerase chain reaction, flow cytometry, and western blotting with anti-P5 antibodies (Supplementary Figure 2). We did not manage to generate a Δ*ompP5* mutant in NTHi P652. This strain has been reported to be resistant to genetic manipulation [35]. Attempts to generate *ompP5-*transcomplemented mutants were not successful, hence they are not included in the current study.

### Binding of Vitronectin to Bacteria

The assay was performed by flow cytometry as previously described [30]. Bacteria (5 × 10^7^ colony forming units [CFU]) from mid-log phase were resuspended in phosphate-buffered saline–1% bovine serum albumin and incubated with human vitronectin (Invitrogen), or with heat-inactivated normal human serum (NHS) at 37°C for 30 minutes. Bound vitronectin was detected with a mouse anti-human vitronectin monoclonal antibody (mAb) (Invitrogen) and a FITC-conjugated goat anti-mouse pAb (BioRad). Samples were analyzed by a CytoFlex flow cytometer (Beckman Coulter) and FlowJo software (Becton Dickison).

### Bacterial Serum Resistance Assay

A serum bactericidal assay was performed as described [29]. Bacteria from mid-log phase (1.5 × 10^5^ CFU/mL) were preincubated with or without 100 nM of purified human vitronectin (Invitrogen) for 30 minutes at 37°C. The sample mixture was then washed, pelleted, and resuspended in assay buffer, followed by incubation with indicated percentages of normal human serum (NHS). At every fifth minute, 10 µL of bacterial suspension was plated on chocolate agar and incubated for 16 hours before colony enumeration on ProtoCOL3 (Synbiosis). All values were adjusted to time zero (T_0min_ × 100). Heat-inactivated NHS was included as a negative control (data not shown).

### Bioinformatic Analysis

Protein sequence of P5 from NTHi 3655 (GenBank accession number, CAH0451603), KR271 (CAH0449785), KR317 (PV387703), and P652 (AXP36880) were aligned using Clustal Omega. Regions corresponding to loops 1 to 4 were identified as previously described [7, 36]. Peptides for loops 1 to 4 of P5 of each strain (NTHi 3655, KR271, KR317, and P652) were synthesized from Genscript Biotech for enzyme-linked immunosorbent assay (ELISA) (Supplementary Table 2).

### Enzyme-Linked Immunosorbent Assay

Direct interactions of vitronectin with loops 1 to 4 of P5 were analyzed by ELISA [29]. Synthetic peptides (100 nM) were immobilized overnight on MaxiSorb microtiter plates (Nunc, ThermoFisher). Plates were washed and added with 20 nM of purified human vitronectin (Invitrogen), followed by 1 hourof incubation at room temperature. After washing, mouse anti-human vitronectin mAb (Invitrogen) followed by horseradish peroxidase-conjugated rabbit anti-mouse (Dako) were used to detect peptide-bound vitronectin.

### Statistical Analyses

We used GraphPad Prism 10.0 for statistical analyses. One- or 2-way ANOVA tests were used and differences were considered statistically significant at *P* < .05.

## RESULTS

### Nontypeable *H. influenzae* Strains Bind Vitronectin at Variable Levels and in a Dose-Dependent Manner

**Figure 1.**
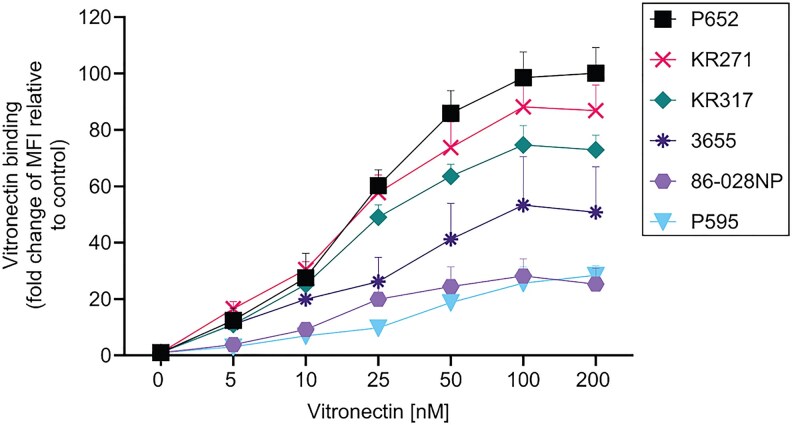
Nontypeable *Haemophilus influenzae* (NTHi) binds vitronectin in a strain-dependent manner. Bacterial strains NTHi P652, KR271, KR317, 3655, 86-028NP, and P595 were incubated with increasing concentrations of purified human vitronectin (5–200 nM) and analyzed by flow cytometry. Surface bound vitronectin was detected by mouse anti-human vitronectin mAb and fluorescein isothiocyanate-conjugated goat anti-mouse polyclonal antibody. Binding to vitronectin is shown as fold change of median fluorescence intensity (MFI) relative to control (bacteria only in the absence of vitronectin). Data represent mean values of 3 independent experiments and error bars indicate standard deviations.

We have previously demonstrated that the majority of NTHi clinical isolates bind vitronectin [29]. In the present study, 6 different NTHi strains were selected for detailed analysis: isolates of COPD (NTHi P652 and P595), invasive disease (KR271), otitis media (3655 and 86-028NP), and bronchitis (KR317) [4, 26, 31–33]. To examine the vitronectin-binding kinetics of NTHi, we incubated the various NTHi strains with increasing concentrations (5–200 nM) of purified human vitronectin (Figure 1 and Supplementary Figure 3). All strains showed saturation in vitronectin binding at concentrations of ≥ 100 nM, although, as expected the binding levels varied between strains. Notably, NTHi P652 showed the strongest binding to vitronectin, followed by KR271, KR317, and 3655. In contrast, NTHi 86-028NP and P595 exhibited the weakest vitronectin binding among the strains tested.

### P5 that was Heterologously Expressed at the Surface of *E. coli* Promotes Vitronectin Binding

**Figure 2.**
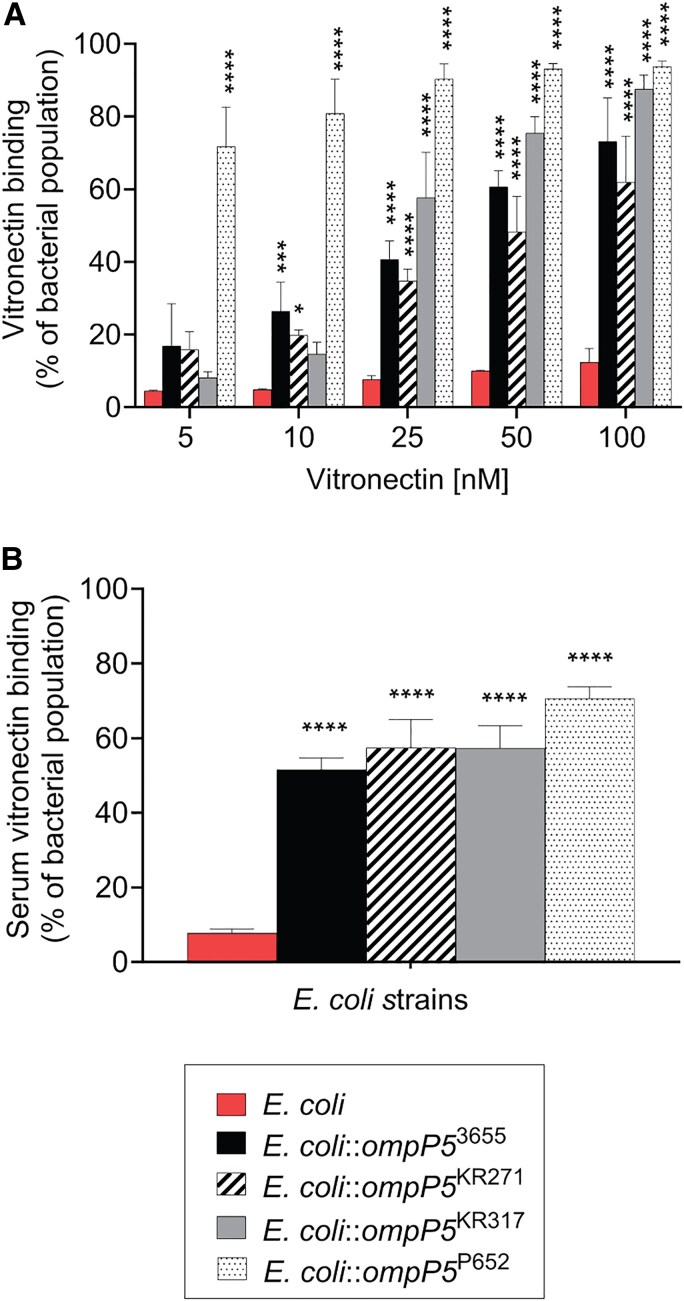
*Escherichia coli* expressing P5 binds vitronectin. *A,* Binding of purified vitronectin to *E. coli* BL21(DE3) expressing P5 from different nontypeable *Haemophilus influenzae* strains (denoted as *E. coli*::*ompP5*^3655^, *E. coli*::*ompP5*^KR271^, *E. coli*::*ompP5*^KR317^, *E. coli*::*ompP5*^P652^). Bacteria were incubated with purified human vitronectin at increasing concentrations (5–100 nM) and binding was detected by flow cytometry. Incubation of bacteria in the absence of vitronectin was included as a negative control. *B*, P5-dependent binding of vitronectin directly from normal human serum (NHS). Strains of *E. coli* expressing P5 variants were incubated with 10% NHS and bound vitronectin was detected by flow cytometry. *A* and *B,* Vitronectin was detected by a mouse anti-human vitronectin mAb and a secondary fluorescein isothiocyanate-conjugated goat anti-mouse polyclonal antibody. The negative control *E. coli* refers to *E. coli* BL21(DE3) only. Thirty thousand events were analyzed for each sample. The vitronectin-binding population (positive population) was gated at 5% of the background binding population in the control (bacteria without vitronectin incubation and was used as antibody background control). Mean values of 3 independent experiments are shown and error bars indicate standard deviations. Statistically significant differences between control *E. coli* and *E. coli-*expressing P5 were calculated by 2-way ANOVA. *P < .05, **P < .01, ***P < .005, ****P < .001.

The OMP P5 is not conserved among NTHi strains, especially at the N-terminal extracellular structure that interacts with host proteins [6, 7, 9]. To characterize the interaction of P5 with vitronectin, we recombinantly expressed P5 from NTHi 3655, KR271, KR317, and P652, respectively, on the surface of *E. coli* BL21(DE3) (Supplementary Figure 4) [12]. Importantly, the expression host *E. coli* BL21(DE3) does not bind vitronectin, which enabled us to study the impact of P5 in the absence of other vitronectin-binding proteins [24, 29]. When incubated with different concentrations of purified human vitronectin (5–100 nM), *E. coli* BL21(DE3) strains expressing P5 variants (*E. coli::ompP5*) showed an increased vitronectin-binding population in a dose-dependent manner, compared to the expression host only (Figure 2*A* and Supplementary Figure 5).

Based on the interaction with purified vitronectin (Figure 2*A*), we further assessed the ability of *E. coli::ompP5* to bind vitronectin directly from NHS [16, 17]. We preincubated bacteria with NHS and bacterial surface-bound vitronectin was analyzed by flow cytometry. As shown in Figure 2*B*, the vitronectin-binding population of *E. coli::ompP5* expressing P5 derived from the different NTHi strains increased compared to *E. coli* only (Figure 2*B*). Thus, the same binding phenotype was observed for the P5 variants, regardless of whether purified vitronectin or vitronectin from NHS was used as a bait. Interestingly, among all the *E. coli-ompP5* strains tested, we noticed that *E. coli::ompP5*^P652^ showed the highest binding to vitronectin, even at the lowest concentration of purified vitronectin (5 nM) (Figure 2). This is in good accordance with the high vitronectin-binding phenotype exhibited by wild-type NTHi P652 in Figure 1. Taken together, our data indicate that P5 is a novel vitronectin-binding protein in NTHi.

### Deletion of P5 From NTHi Results in Reduced Bacterial Vitronectin Binding

To investigate the impact of P5 in vitronectin binding of NTHi while in the presence of other endogenous vitronectin receptors (PE, PF, and P4), we deleted the P5 gene (*ompP5*) from NTHi 3655, KR271, and KR317 [12, 27–30]. Then, the NTHi 3655Δ*ompP5*, KR271Δ*ompP5,* and KR317Δ*ompP5* mutants were subjected to a direct binding assay with vitronectin and compared to their parental wild-type strains.

**Figure 3.**
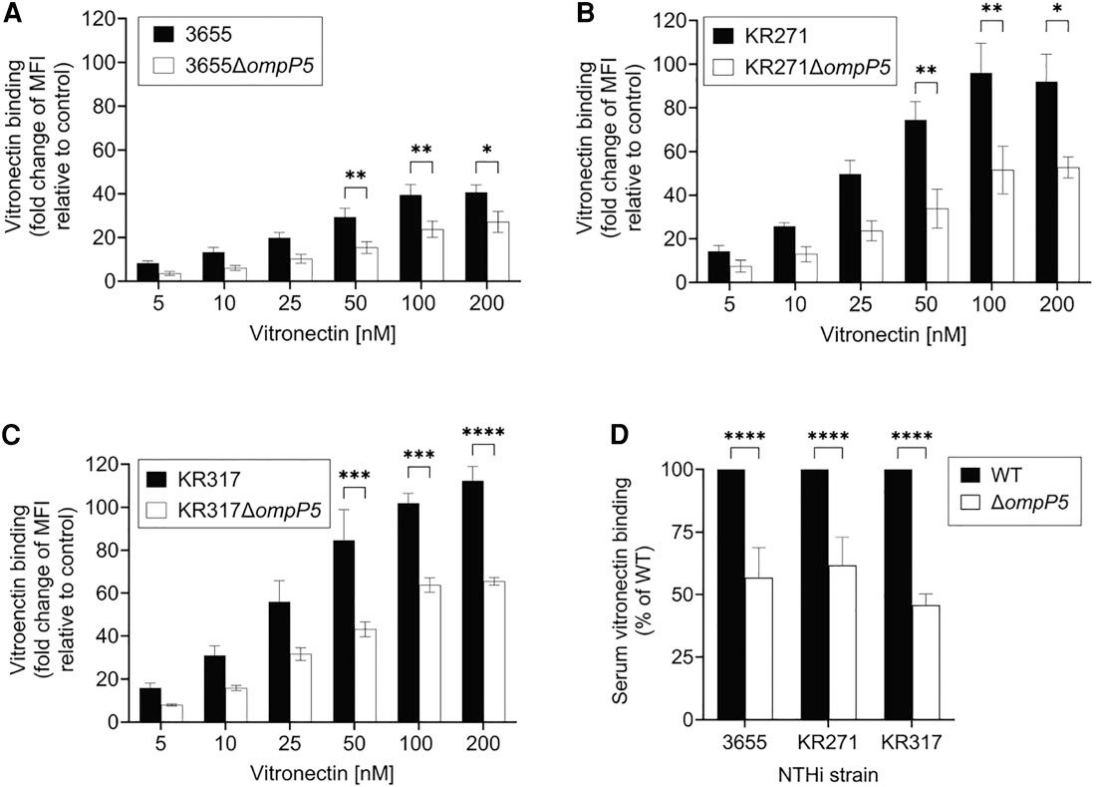
OMP P5 is important for vitronectin binding in NTHi. WT and Δ*ompP5* NTHi mutants devoid of P5 were incubated with purified human vitronectin or NHS. *A–C*, WT strains (black bars) of NTHi 3655 (*A*), KR271 (*B*), and KR317 (*C*) bound more vitronectin compared to their P5-deficient counterparts (Δ*ompP5*) (white bars) at all concentrations tested. Vitronectin binding is presented as the fold change of MFI in relation to bacteria only (incubated in the absence of vitronectin as a negative control). *D*, NTHi strains were incubated with 10% NHS, followed by measurement of vitronectin binding. Vitronectin binding of NTHi WT was normalized to 100% for each strain and binding of vitronectin to NTHi Δ*ompP5* mutants is presented as percentage binding relative to WT. *A–D,* Surface bound vitronectin was detected by mouse anti-human vitronectin monoclonal antibody and fluorescein isothiocyanate-conjugated goat anti-mouse polyclonal antibody. Mean values of 3 independent experiments are shown and error bars indicate standard deviations. Statistically significant differences between WT and Δ*ompP5* mutant were calculated by 2-way ANOVA. *P < .05, **P < .01, ***P < .005, ****P < .001. Abbreviations: MFI, median fluorescence intensity; NHS, normal human serum; NTHi, nontypeable *Haemophilus influenzae;* OMP, outer membrane protein; WT, wild type.

Deletion of P5 resulted in significantly decreased vitronectin binding for the Δ*ompP5* mutants compared to their wild-type counterparts (Figure 3*A–C* and Supplementary Figure 6). The same vitronectin-binding phenotype was observed when NHS was used (Figure 3*D*), where NTHi Δ*ompP5* mutants only retained between 45% and 62% of their vitronectin-binding capacity compared to their respective parental wild types. In conclusion, our data suggest that in addition to the well-characterized OMPs PE, PF, and P4, P5 also serves as an important vitronectin receptor for NTHi.

### Recruitment of Vitronectin via P5 Protects Bacteria From Complement-Mediated Killing

**Figure 4.**
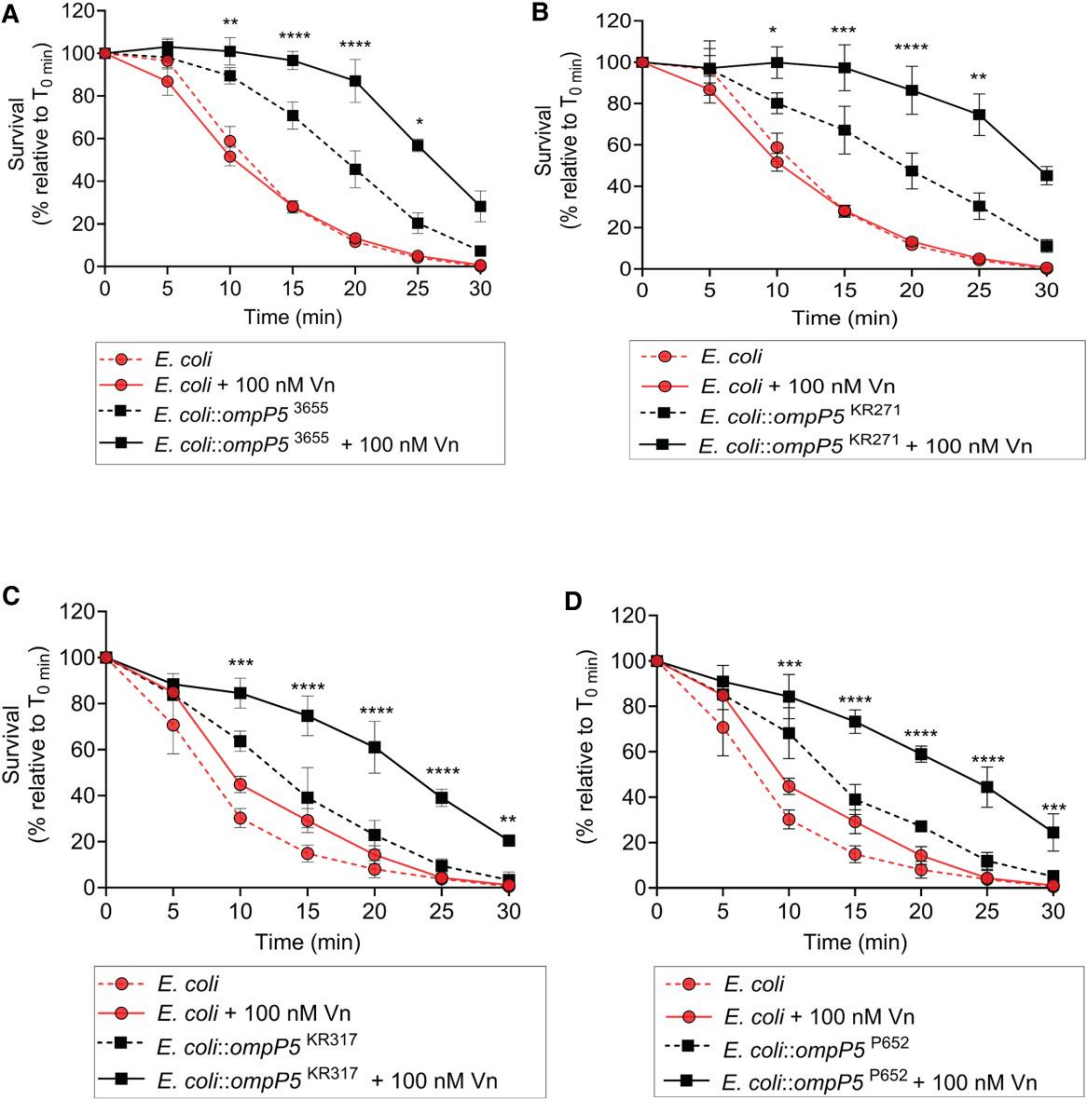
P5-dependent Vn binding promotes serum survival in *Escherichia coli* expressing P5. *E. coli* expressing P5 variants derived from NTHi 3655, KR271, KR317, and P652 were incubated with 1% NHS with or without 100 nM Vn preincubation. *A–C,* Serum survival of *E. coli::ompP5*^3655^ (*A*), *E. coli::ompP5*^KR271^ (*B*), *E. coli::ompP5*^KR317^ (*C*), and *E. coli::ompP5*^P652^ (*D*) was determined at different time points. Control *E. coli* only was included as negative control in (*A–D*). Serum survival is shown as a percentage of survival, which was calculated as colony forming units relative to time zero (T_0min_ × 100). Mean values from 3 independent experiments are shown and error bars indicate standard deviations. Statistically significant differences in serum survival between bacteria preincubated with Vn and without preincubation were calculated by 2-way ANOVA. *P < .05, **P < .01, ***P < .005, ****P < .001. Abbreviations: NHS, normal human serum; NTHi, nontypeable *Haemophilus influenzae*; Vn, vitronectin.

Since vitronectin inhibits the formation of the MAC at the terminal step of the complement cascade, the recruitment of vitronectin to the bacterial surface enhances bacterial resistance against complement-mediated killing [16]. To investigate whether the P5-dependent vitronectin binding could also promote bacterial serum resistance, we first examined *E. coli* expressing the different P5 variants in a serum killing assay, with or without preincubation with vitronectin. Of note, preincubation of bacteria with exogenous vitronectin enabled the serum killing assay to be carried out with native NHS. As shown in Figure 4*A–D*, all *E. coli*::*ompP5* strains (expressing NTHi-P5^3655^, -P5^KR271^, -P5^KR317^, or -P5^P652^) tested were killed more slowly compared to the *E. coli* host only. Because NTHi strains were saturated with 100 nM of vitronectin (Figure 1), this concentration (100 nM) was used for preincubation with vitronectin. Addition of vitronectin further increased the serum survival in *E. coli*::*ompP5* strains. In contrast, vitronectin supplementation, as expected, had no effect on the serum survival of control *E. coli*.

**Figure 5.**
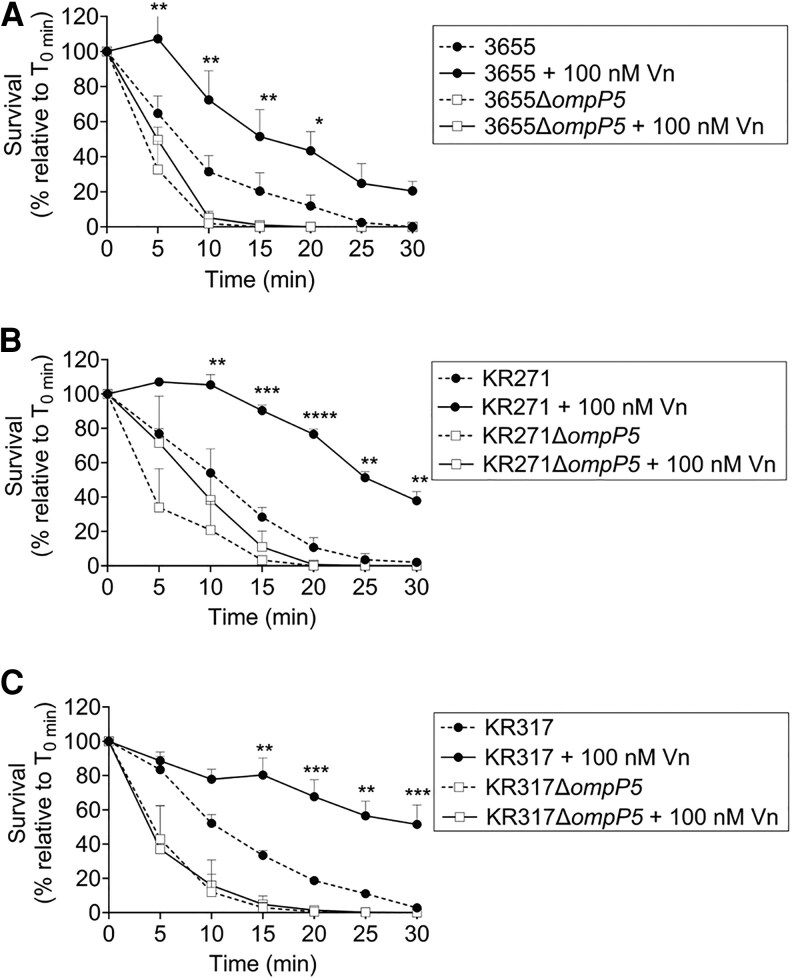
P5-dependent binding of Vn promotes serum survival in NTHi. *A–C*, P5-deletion mutants of 3655Δ*ompP5* (*A*), KR271Δ*ompP5* (*B*), and KR317Δ*ompP5* (*C*) and their parental wild-type strains were subjected to NHS killing, with or without preincubation with 100 nM purified Vn. The concentration of NHS used was strain dependent and was optimized for each strain, that is 2.5%, 7.5%, and 30% of NHS for NTHi 3655, KR271, and KR317, respectively. Percentage survival was determined at different time points and calculated as colony forming units relative to time zero (T_0min_ × 100). Mean values of 3 independent experiments are shown and error bars indicate standard deviations. Statistically significant differences in serum survival between bacteria with or without Vn preincubation were calculated by 2-way ANOVA. *P < .05, **P < .01, ***P < .005, ****P < .001. Abbreviations: NHS, normal human serum; NTHi, nontypeable *Haemophilus influenzae*; Vn, vitronectin.

To analyze the role of P5 in NTHi serum resistance, NTHi Δ*ompP5* mutants of 3655, KR271, and KR317 and their respective wild-type strains, were incubated in the presence of NHS. The P5-deficient NTHi mutants were all more sensitive to NHS and killed more rapidly compared to the wild-type bacteria carrying P5 (Figure 5*A–C*). The supplementation with vitronectin (100 nM) did not improve serum survival of mutants. Wild-type bacteria did, however, survive better after utilizing vitronectin added to the reaction mixtures. Taken together, our data show that P5 plays a significant role in bacterial resistance against complement-mediated killing through the recruitment of vitronectin to the bacterial surface.

### The Extracellular Loop 2 of P5 Is Involved in the Interaction With Human Vitronectin

**Figure 6.**
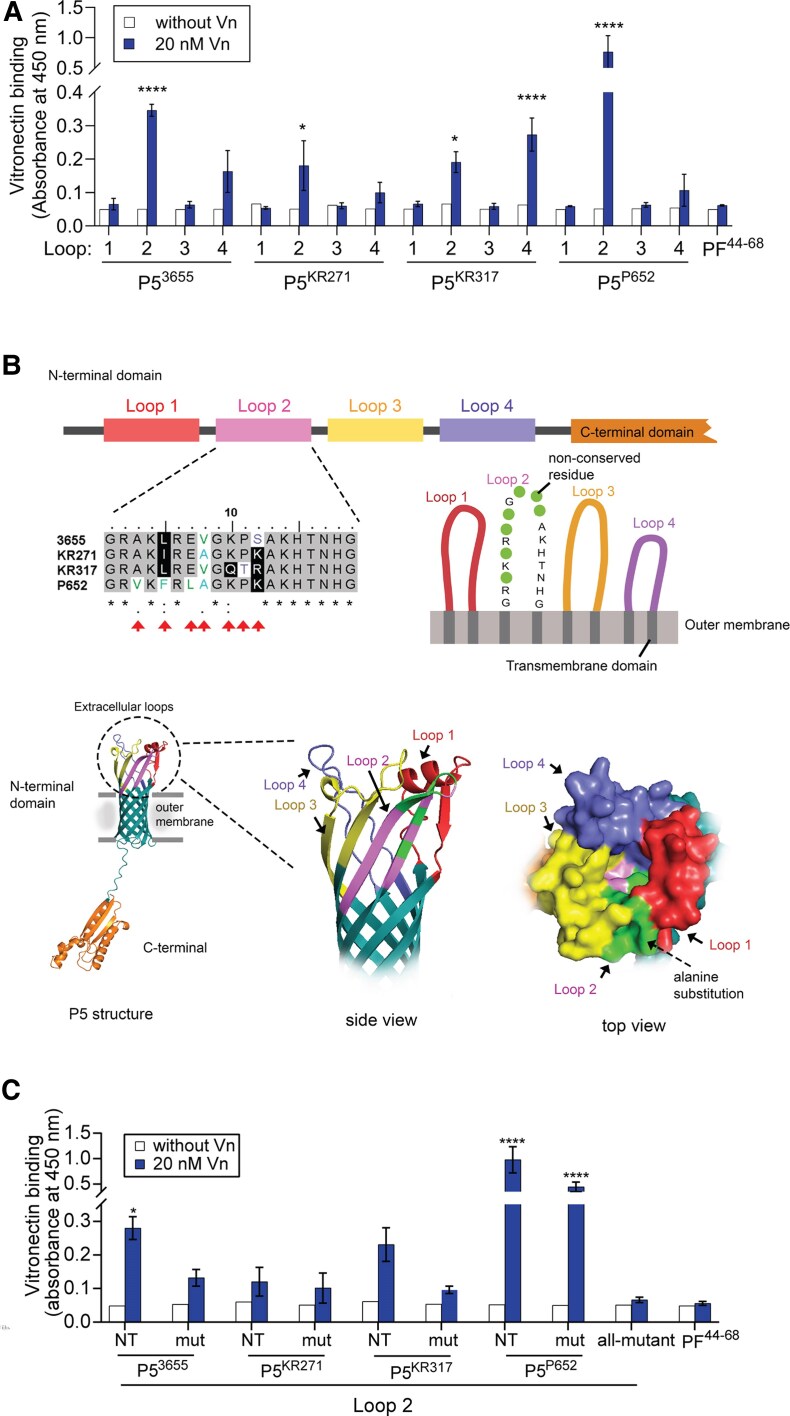
The NTHi P5 loop 2 interact with Vn. *A*, Binding of Vn to the extracellular loops 1 to 4 of P5 from NTHi 3655, KR271, KR317, and P652 was analyzed by ELISA. Peptide PF^44–68^, which does not bind Vn, was included as a negative control [29]. White bars indicate control without Vn whereas black bars indicate addition of 20 nM Vn*. B*, Multiple sequence alignment of loop 2 of P5 from NTHi 3655, KR271, KR317, and P652 (above). Nonconserved amino acids among loop 2 variants are indicated with red arrows; asterisk indicates identical residues; colon indicates similar residue; and period indicates semisimilar residue. Diagrammatic representation of the predicted membrane topology of P5 loop 2 showing amino acid positions with nonconserved residues (green circles, upper right). The nonconserved residues that are specific to each loop 2 variant were substituted with alanine to generate different mutated peptides of loop 2 (Supplementary Table 2). Below is shown a cartoon of the protein structure of P5. The N-terminal extracellular loops and C-terminal peptidoglycan-binding domain of P5 are shown (left). The 3-dimensional structure shown is based on the AlphaFold model of P5^3655^ (Uniprot number, A0A0H3PCS3). Enlarged side and top view of the extracellular loops of P5 are shown (center and right). The location of the nonconserved residues in loops 2 are indicated in green. *C,* Binding of Vn to the native and mutated loop 2 of each P5 variants (ie, loop 2^L5A-V8A-S12A^ for P5^3655^, loop 2^I5A^ for P5^KR271^, loop 2^L5A-V8A-Q10A-T11A-R12A^ for P5^KR317^, and loop 2^V3A-F5A-L7A^ for P5^P652^) as analyzed by ELISA. Loop 2^all-mutant^ has alanine substitution in all the nonconserved positions (3rd, 5th, 7th, 8th, 10th, 11th, and 12th). The mean values of 3 independent experiments are shown and error bars indicate standard deviations. Statistically significant differences in Vn binding between extracellular loops of P5 variants and control peptide PF^44–68^ (*A*), between variants-specific native and mutated loop 2, and between native loop 2 of each variant and loop 2^all-mutant^ (*C*) were calculated by a 2-way ANOVA. *P < .05, ****P < .001. Abbreviations: ELISA, enzyme-linked immunosorbent assay; mut, mutated; NT, native; NTHi, nontypeable *Haemophilus influenzae*; Vn, vitronectin.

The extracellular surface-exposed loops of P5 have been the binding targets for many host proteins [6, 7, 9, 37]. We hypothesized that the P5 loops might also be the binding sites for human vitronectin. To test this, a series of peptides corresponding to loops 1 to 4 of P5 from NTHi 3655, KR271, KR317, and P652 were analyzed for their vitronectin binding in ELISA (Supplementary Table 2 and Figure 6*A*). Due to the difficulty in producing soluble P5 under native condition, synthetic peptides have been widely used as a model for P5 loops [37–40]. We observed that loop 2 followed by loop 4 for P5^3655^, P5^KR271^, and P5^P652^ constantly showed higher binding to vitronectin compared to their respective loop 1 and 3 (Figure 6*A*). However, for P5^KR317^, loop 4 exhibited the strongest binding followed by loop 2. Compared to other loop 2 variants, the P5^P652^-derived loop 2 had the strongest binding. This was consistent with the strong vitronectin binding phenotype of NTHi P652 and *E. coli* expressing P5^P652^, compared to the other strains tested (Figure 1 and Figure 2). Our data suggested that loop 2 of P5 is the main binding site for vitronectin while loop 4 could be an alternative site.

We then further investigated whether there was any specific motif(s) or residue(s) that might contribute to the variation in binding between the loop 2 variants. Sequence alignment of loop 2 variants revealed that there were 7 amino acid positions (ie, third, fifth, seventh, eight, and 10th to 12th) that are not conserved (Figure 6*B*). Most of the nonconserved residues are either nonpolar or uncharged. It was previously reported that amino acid at the positions of fifth, seventh, eighth, and 12th of loop 2 were prone to residue change in persistent strains of NTHi isolated from COPD patients [9]. Because the loop 2-vitronectin interaction is still unexplored, we first mutated residues that are unique for each loop 2 variant (Figure 6*B* and Supplementary Table 2) and analyzed their vitronectin binding. As shown in Figure 6*C*, replacing residues V3, F5, and L7 with alanine partially reduced the vitronectin binding of the mutated loop 2 of P5^P652^ (loop 2^V3A-F5A-L7A^) compared to its native loop 2. Similarly, mutating 3 residues (L5, V8, and S12) and 5 residues (L5, V8, Q10, T11, and R12) in loops 2 of P5^3655^ (loop 2^L5A-V8A-S12A^) and P5^KR317^ (loop 2^L5A-V8A-Q10A-T11A-R12A^) caused reduction in binding compared to their respective native loop 2. For P5^KR271^, we did not observe any difference in binding between the native loop 2 and the mutated loop 2^I5A^ that had single alanine substitution at I5. In general, multiple residues mutagenesis instead of single mutation constantly resulted in reduced binding.

We also generated a mutant peptide of loop 2 (loop 2^all-mutant^) that had alanine substitutions in all the nonconserved positions (third, fifth, seventh, eighth, 10th, 11th, and 12th) to understand their total effect in vitronectin binding (Figure 6*B*). The mutagenesis significantly reduced the binding of the loop 2 mutant compared to both the mutated loop 2 variants with fewer residue replacements and the native loop 2 of all P5 variants (Figure *6C*). In conclusion, our data suggest that P5 variants bind vitronectin via loop 2 and that the variability in binding phenotype could be attributed to sequence diversity of loop 2.

## DISCUSSION

In this study, we showed that vitronectin is another complement regulator targeted by P5 for increased serum survival in *H. influenzae*. Because P5 is not conserved in NTHi, we selected P5 variants from vitronectin-binding strains for further study (Figure 1). The protein sequences of the N-terminal extracellular regions of P5^3655^, P5^KR271^, P5^KR317^, and P5^P652^ are not conserved, with sequence identities of 87.2%–94.8% and similarities of 90.6%–96.6% (Supplementary Figure 7). It is plausible that these differences contribute to the variation in vitronectin-binding affinity among the P5 variants when expressed on the surface of the heterologous host, *E. coli* BL21 (DE3) (Figure 2 and Supplementary Figure 4). Moreover, protein sequence variation in P5 is also associated with protein expression levels that might also ultimately affect vitronectin binding, compared to P4, PE, and PF that are more conserved and similarly expressed among NTHi strains [29, 30, 41].

In contrast to encapsulated strains of *H. influenzae,* NTHi strains are not clonal [42]. Deletion *ompP5* from NTHi strains, 3655, KR271, and KR317, however, resulted in reduced vitronectin binding (Figure 3). Partial reduction (approximately 30%–50%) in vitronectin binding was observed in the Δ*ompP5* mutants despite the presence of other vitronectin receptors (P4, PE, and PF) (Supplementary Figure 8). This could be attributed to the topology of P5 as the major OMP in the wild-type strains [8]. The combined observations from both models of *ompP5*-knock-in *E. coli* (as a heterologous host) and NTHi P5-deficient mutants, strongly confirmed the P5 variants (P5^3655^, P5^KR271^, P5^KR317^, and P5^P652^) as vitronectin receptors.

Complement regulators hijacked at the bacterial surface need to be functional to protect bacteria from host complement-mediated killing. The P5-vitronectin complex functionality in serum resistance was supported by 2 experimental findings (Figure 4 and Figure 5). Firstly, *E. coli* expressing P5 variants survived better in the serum killing assay when presupplemented with exogenous vitronectin compared to *E. coli* only; and, secondly, NTHi Δ*ompP5* mutants did not respond to the addition of vitronectin yet remained more sensitive to complement-mediated killing compared to their wild-type strains.

P5 of NTHi 3655 and KR271 bind C4BP at loop 2, while loop 1 and 2 of P5 from NTHi R2866 are the binding sites for FH [12, 13]. Our current study revealed that P5 binds vitronectin mainly via loop 2 (Figure 6). Loop 2 variants have the highest isoelectric point (pI) of 11.1–12.03 compared to other loops (Supplementary Table 2). On the other hand, vitronectin has a lower pI value of 5.55, including its hemopexin repeats (pI = 4.41–5.08) [43, 44]. This causes loop 2 and vitronectin to be positively and negatively charged, respectively, when exposed to physiological pH (approximately 7.40) [45, 46]. We speculate that the opposite charges between loop 2 and vitronectin might provide electrostatic forces, enabling a higher binding affinity between loop 2 and vitronectin (Figure 6*A*). While alanine substitution, which mainly replaces the nonpolar amino acids at nonconserved positions, did not alter the pI value of the mutated peptides, the mutagenesis, however, impaired the vitronectin-binding of the mutant peptides (Figure 6*C*). It is plausible that the nonconserved amino acid residues might also be involved in ligand binding by affecting the conformation of loop 2 and hence the accessibility to vitronectin (Figure 6*B*). The amino acid sequence diversity and their positions in loop 2 ultimately contributes to variation in binding between variants. It has been reported that PE and PF of NTHi bind vitronectin via nonionic and ionic interactions, respectively [24, 29]. However, we could not rule out the mechanism(s) of protein-protein interaction (ie, electrostatic forces, hydrogen bonding, or the hydrophobic effect) occurred between vitronectin and P5, and thus this needs to be further studied.

Here we postulate that the sequence diversity (especially the loop 2) as well as the variability in P5 expression could be the mechanisms underlying the differences in vitronectin binding between NTHi strains. It is not unusual to have nonconserved-binding sites within the same receptor family. For example, the vitronectin-binding site of ubiquitous surface protein A (UspA2) variants in *Moraxella catarrhalis* is not conserved but rather highly hypervariable at the head domain [47]. The extracellular loops of P5 have been well studied regarding their sequence diversity and immunogenicity [6, 7, 9, 48]. Unlike loop 4, which is more conserved, loop 2 is more diverse among NTHi strains and not as immunogenic as loops 3 and 4. This could be a pathoadaptation strategy used by NTHi for persistent infection, enabling the pathogen to hijack host complement regulators via loop 2 of P5 while avoiding host immune attacks [6, 9].

The expression of P5 as the major OMP with multifunctional loops provides an advantage for NTHi to concurrently hijack various complement regulators, including C4BP and FH, maximizing resistance against host complement-mediated killing. A competition assay showed that despite sharing the same binding site on P5, which is in loop 2, vitronectin, C4BP, and FH do not compete with each other in binding to P5 (Supplementary Figure 9). The role of the OmpA family proteins in recruiting host complement regulators for increased serum resistance, that is FH and C4BP, has also been reported in *Acinetobacter baumannii* and *E. coli* K1, respectively [49, 50]. In conclusion, our work is the first study showing P5 as a novel vitronectin receptor of NTHi, with its interaction contributing to bacterial survival against complement-mediated killing. Our findings have also highlighted the sophisticated OmpA family proteins-associated virulence strategy utilized by airway pathogens to combat host innate defense, as exemplified by OMP P5 of NTHi through its ability to employ different complement regulators.

## Supplementary Material

jiaf489_Supplementary_Data

## Supplementary Data

Supplementary materials are available at *The Journal of Infectious Diseases* online (http://jid.oxfordjournals.org/). Supplementary materials consist of data provided by the author that are published to benefit the reader. The posted materials are not copyedited. The contents of all supplementary data are the sole responsibility of the authors. Questions or messages regarding errors should be addressed to the author.
