# Supplementary material for: The Major Outer Membrane Protein P5 Binds Vitronectin to Mediate Serum Resistance in Nontypeable Haemophilus influenzae
Source: J Infect Dis. 2025 Sep 22;232(6):e981–91. doi: 10.1093/infdis/jiaf489 (PMC12718013; doi:10.1093/infdis/jiaf489)
Supplement: jiaf489_Supplementary_Data [file jiaf489_supplementary_data.docx]

**Supplemental Material**

**The major outer membrane protein P5 binds vitronectin to mediate serum resistance in non-typeable Haemophilus influenzae**

Sandra Jonsson^1^, Martina Janoušková^1^, Vaishnavi Venkatesh Rao^1^, Junkal Garmendia^2,3^, Yu-Ching Su^1^, and Kristian Riesbeck^1†^

^1^Clinical Microbiology, Department of Translational Medicine, Faculty of Medicine, Lund University, Malmö, Sweden. ^2^Instituto de Agrobiotecnología, Consejo Superior de Investigaciones Científicas (IdAB-CSIC)-Gobierno de Navarra, Mutilva, Madrid, Spain. ^3^Centro de Investigación Biomédica en Red de Enfermedades Respiratorias (CIBERES), Madrid, Spain.

^†^Corresponding author:
Clinical Microbiology, Department of Translational Medicine, Faculty of Medicine, Lund University, Inga Marie Nilssons gata 53, SE-214 28, Malmö, Sweden
[kristian.riesbeck@med.lu.se](mailto:kristian.riesbeck@med.lu.se)

**
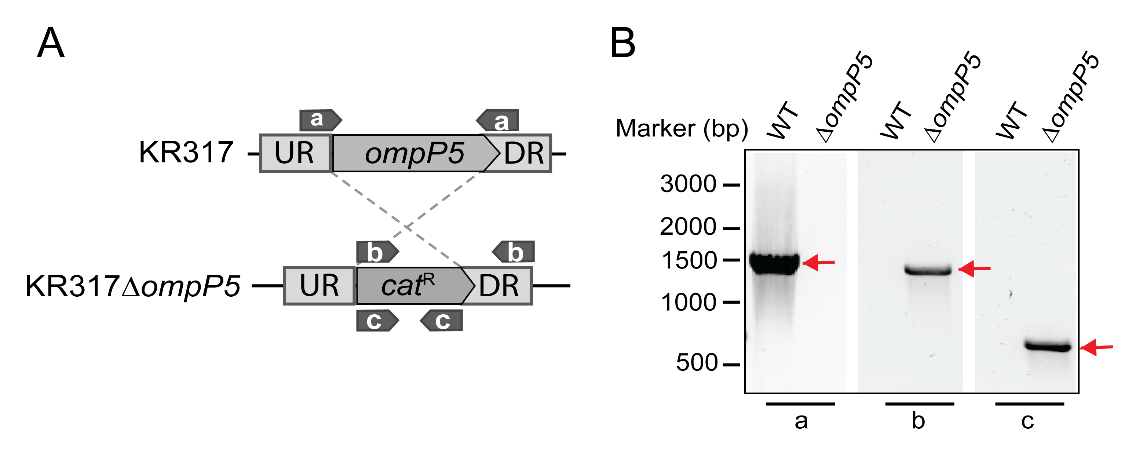
**

**Figure S1.** Construction of NTHi KR317∆*ompP5* isogenic mutant. *A*, Schematic representation showing the replacement of P5-encoding gene (o*mpP5*) with a chloramphenicol acetyltransferase gene (*cat*) in KR317. *B,* Verification of *cat* replacement and *ompP5* deletion in KR317∆*ompP5* by polymerase chain reaction (PCR). PCR products were analyzed on 1% agarose gel. Gels were documented on a ChemiDoc^™^ XRS+ System (Bio-Rad) and analysed with the Image Lab^™^ Software (Bio-Rad). The position of primer pairs used for PCR verification are indicated by black arrows labelled as a, b, and c in panel *A*, Amplicon of primer pair “a” is *ompP5*, present only in the wild type KR317 (WT) but absent in the KR317∆*ompP5* (∆*ompP5*). Primer pairs “b” and “c” are amplifying the regions of UR-*cat* and *cat*-DR, respectively, that are only present in the *omp*A mutant but not in the wild type (WT) KR317. PCR products are indicated with red arrows. UR, upstream region of *ompP5*; DR-downstream region of *ompP5*.


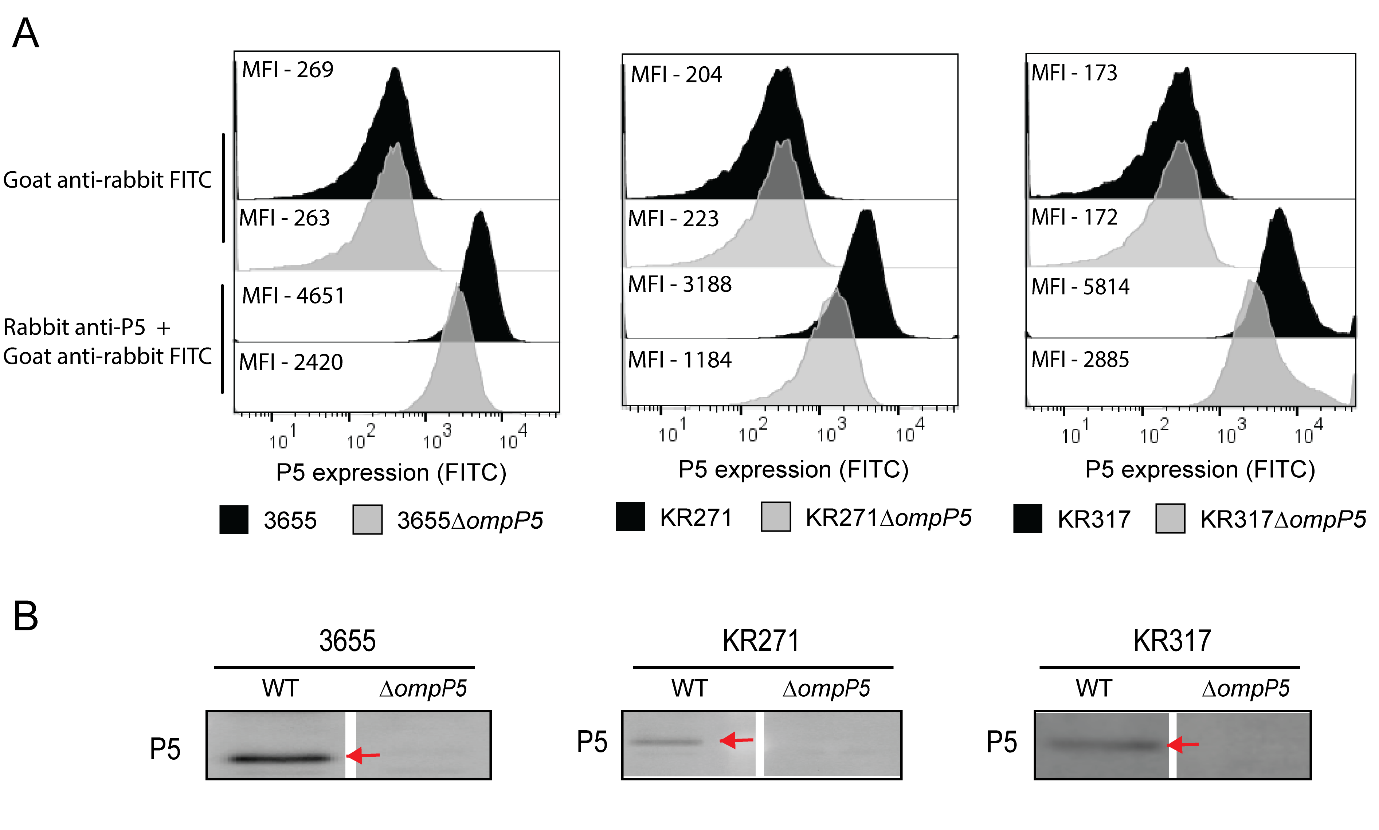


**Figure S2.** Analysis of P5 expression in the wild type and *ompP5*-knockout mutants of NTHi 3655, KR271 and KR317. *A*, Detection of P5 on the surface of wild type and ∆*ompP5* mutant strains by cytometry. A representative histogram of WT (black) and ∆*ompP5* (grey) is shown. Samples were recorded on Cytoflex and data was analysed with FlowJO software. *B,* Western blotting analysis of wild type (WT) and ∆*ompP5* mutants. Immunoblotting signals were documented on ChemiDoc^™^ XRS+ System (Bio-Rad) and analysed with the Image Lab^™^ Software (Bio-Rad). For *A* and *B,* expression of P5 in the wild type and ∆*ompP5* mutant of NTHi 3655 and KR317 was detected with rabbit anti-P5_loop3^3655^ pAb; and P5 expression in the KR271 wild type and ∆*ompP5* isogenic mutant was detected with anti-P5_loop4^3655^ pAbs. Goat anti-rabbit FITC pAb (AbCam) and swine anti-rabbit HRP pAb (Dako) were used as the secondary antibody in *A* and *B,* respectively. Red arrows indicate the western blotting signal corresponding to P5. FITC, fluorescein isothiocyanate; HRP, horseradish peroxidase.

**
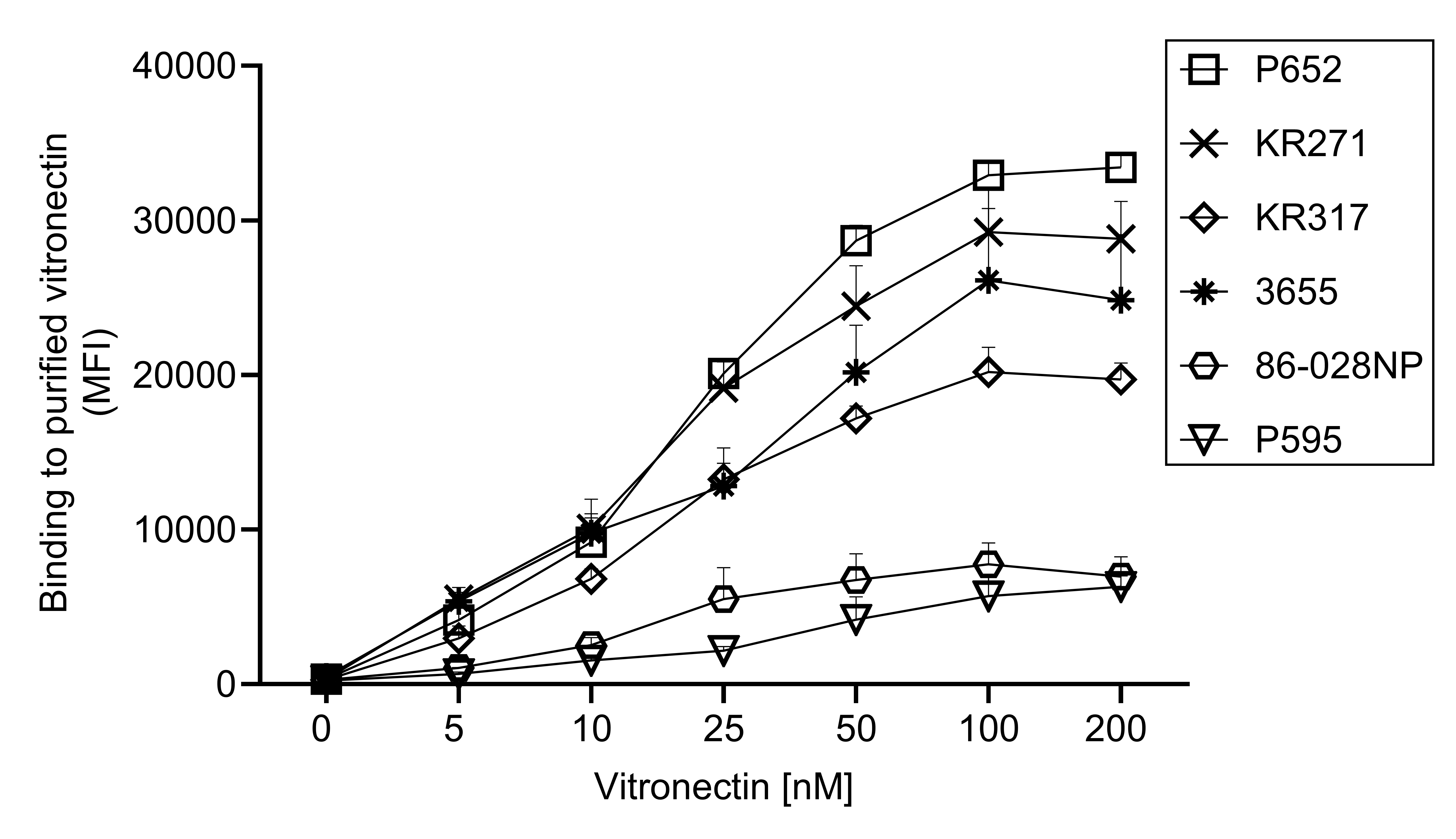
**

**Figure S3:** Median fluorescence intensity (MFI) raw data for Figure 1. Non-typeable *H. influenzae* (NTHi) binds vitronectin in a strain-dependent manner. Bacterial strains NTHi P652, KR271, KR317, 3655, 86-028NP and P595 were incubated with increasing concentrations of purified human vitronectin (5 – 200 nM) and analysed by flow cytometry. Surface bound vitronectin was detected by mouse anti-human vitronectin mAb and FITC-conjugated goat anti-mouse pAb. Binding to vitronectin is shown as MFI. Data represents mean values of three independent experiments and error bars indicate standard deviations.

**
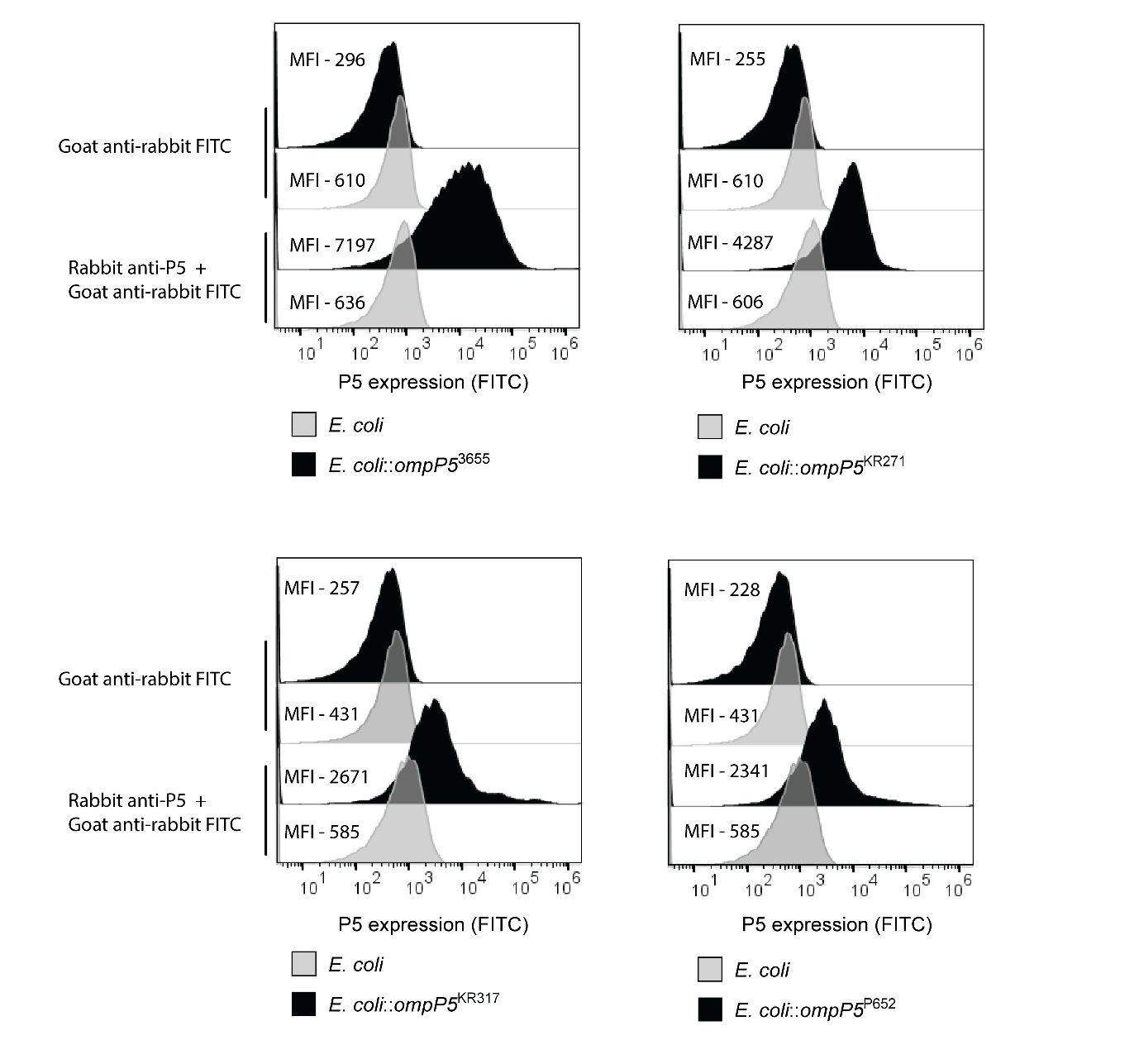
**

**Figure S4.** Flow cytometry analysis of P5 expression on the surface of *E. coli* BL21(DE3)*.* P5 variants derived from NTHi 3655, KR271, KR317 and P652 were expressed on the surface of *E. coli* BL21(DE3) that were later denoted as *E. coli*::*ompP5*^3655^, *E. coli*::*ompP5*^KR271^, *E. coli*::*ompP5*^KR317^ and *E. coli*::*ompP5*^P652^, respectively. Figure shows a representative histogram of P5 expression on the surface of *E. coli* for each P5 variant. Expression of P5 variants from NTHi 3655, KR317 and P652 on the surface of *E. coli* was detected with rabbit anti-P5_loop3^3655^ pAb. Anti-P5_loop4^3655^ pAb was used to detect the expression of P5 variant from KR271 on the surface of *E. coli*. Swine anti-rabbit HRP pAb (Dako) was used as the secondary antibody. Immunoblotting signals were documented on ChemiDoc^™^ XRS+ System (Bio-Rad) and analysed using the Image Lab^™^ Software (Bio-Rad). FITC, fluorescein isothiocyanate; HRP, horseradish peroxidase.

**
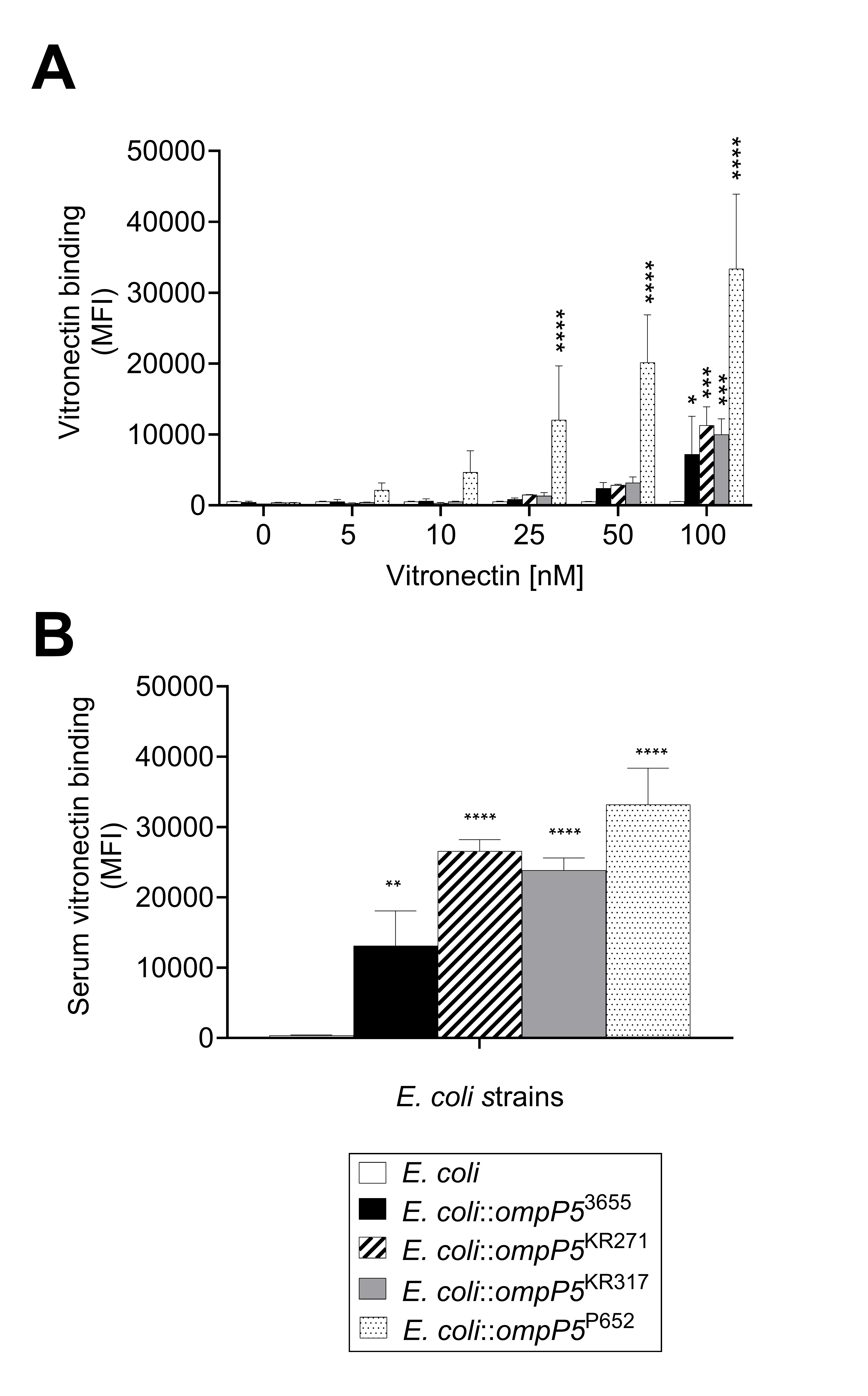
**

**Figure S5:** Median fluorescence intensity (MFI) raw data for Figure 2. *Escherichia coli* expressing P5 binds vitronectin. *A****,*** Binding of purified vitronectin to *E. coli* BL21(DE3) expressing P5 from different NTHi strains (denoted as *E. coli*::*ompP5*^3655^, *E. coli*::*ompP5*^KR271^, *E. coli*::*ompP5*^KR317^, *E. coli*::*ompP5*^P652^). Bacteria were incubated with purified human vitronectin at increasing concentrations (5 – 100 nM) and binding was detected by flow cytometry. Incubation of bacteria in the absence of vitronectin was included as a negative control. *B*, P5-dependent binding of vitronectin directly from normal human serum (NHS). Strains of *E. coli* expressing P5 variants were incubated with 10% NHS and bound vitronectin was detected by flow cytometry. In panels *A* and *B,* vitronectin was detected by a mouse anti-human vitronectin mAb and a secondary FITC-conjugated goat anti-mouse pAb. The negative control *E. coli* refers to *E. coli* BL21(DE3) transformed with empty vector pET16b. Binding to vitronectin is shown as MFI. Mean values of three independent experiments are shown and error bars indicate standard deviations. Statistically significant differences between control *E. coli* and *E. coli-*expressing P5 were calculated by two-way ANOVA. *, *p*<0.05; **, *p*<0.01; ***, *p*<0.005; and ****, *p*<0.001.

**
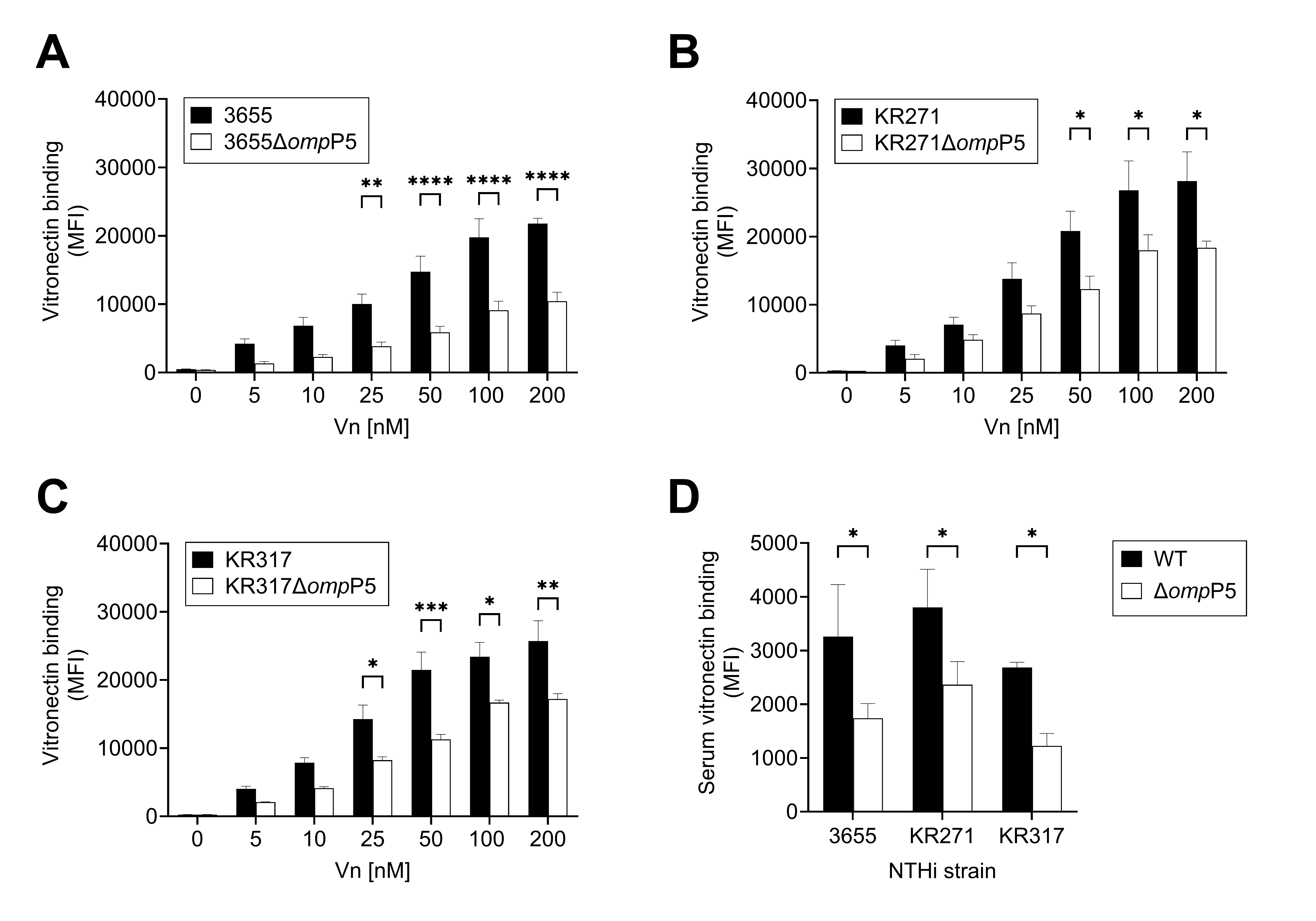
**

**Figure S6:** Median fluorescence intensity (MFI) raw data for Figure 3. OMP P5 is important for vitronectin binding in NTHi. Wild type and Δ*ompP5* NTHi mutants devoid of P5 were incubated with purified human vitronectin or NHS. *A-C*, Wild type (WT) strains (black bars) of NTHi 3655 (*A*), KR271 (*B*) and KR317 (*C*) bound more vitronectin compared to their P5-deficient counterparts (∆*ompP5*) (white bars) at all concentrations tested. In (*D*), NTHi strains were incubated with 10% NHS, followed by measurement of vitronectin binding. Binding to vitronectin is shown as MFI. Surface bound vitronectin in *A-D* was detected by mouse anti-human vitronectin mAb and FITC-conjugated goat anti-mouse pAb. Mean values of three independent experiments are shown and error bars indicate standard deviations. Statistically significant differences between WT and ∆*ompP5* mutant were calculated by two-way ANOVA. *, *p*<0.05; **, *p*<0.01; ***, *p*<0.005; and ****, *p*<0.001


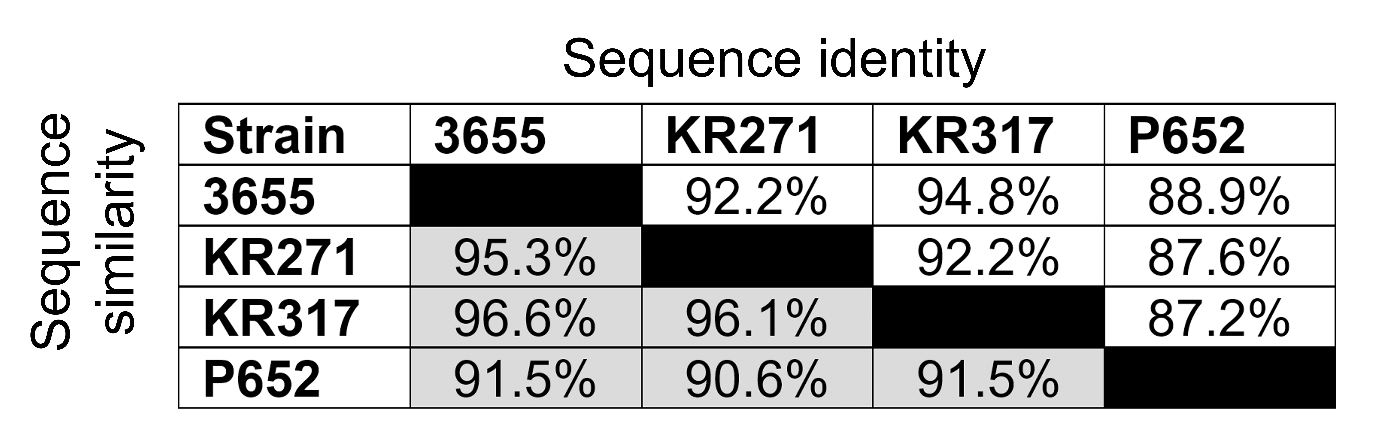


**Figure S7.** Pairwise sequence alignment of the N-terminal extracellular region of P5 variants from NTHi 3655, KR271, KR317 and P652. Program Emboss Needle (https://www.ebi.ac.uk/jdispatcher/psa/emboss_needle) was used to perform global sequence alignment. N-terminal region of P5 variants from NTHi 3655 (GenBank Accession number: CAH0451603), KR271 (CAH0449785), KR317 (PV387703) and P652 (AXP36880) was determined as previously described [1]. Percentage of sequence identity and sequence similarity between two strains are shown in white and grey boxes, respectively.

**Figure S8:** Comparative binding of vitronectin to different vitronectin-binding protein-knockout mutants of NTHi 3655. Wild type NTHi 3655 and isogenic mutants of Δ*ompP5*, Δ*hel*, Δ*hpe* and Δ*hpf* were incubated with 25 nM of vitronectin. Bacterial bound vitronectin was detected with mouse anti-human vitronectin monoclonal antibodies (Thermo Fisher Scientific) and goat anti-mouse IgG-FITC (BioRad). Binding to vitronectin was measured as median fluorescence intensity (MFI) on flow cytometry using CytoFlex flow cytometer and the FlowJo software. Deletion of *ompP5* caused the most reduction in vitronectin binding, compared to wild type and other gene-knockout mutants. Statistically significant differences between wild type and mutant in binding to purified vitronectin were calculated by one-way ANOVA. **, *p*<0.01; ***, *p*<0.005; and ****, *p*<0.001.


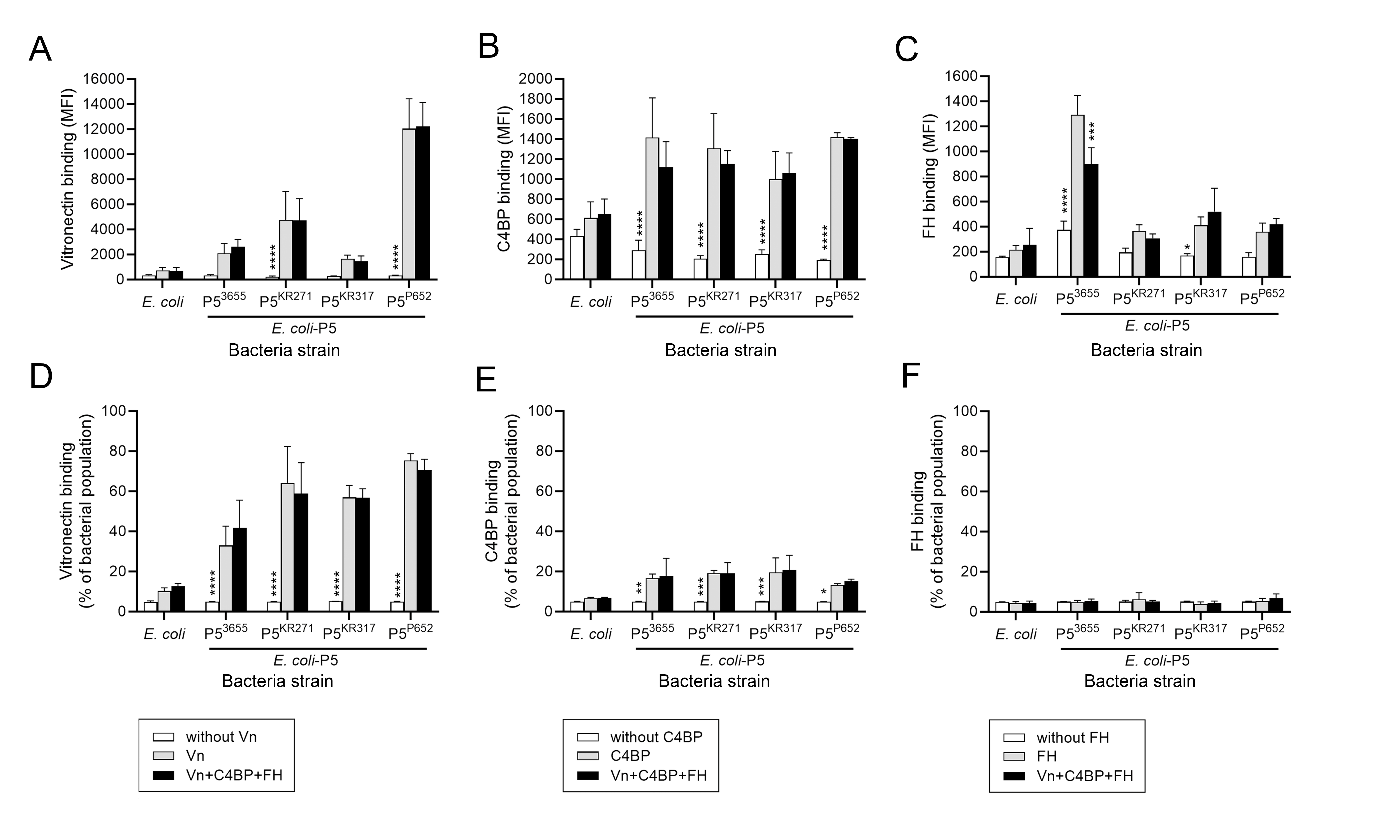


**Figure S9:** Detection of variable complement regulators binding by *E. coli* expressing P5 variants. *A-F, Escherichia coli* expressing all P5 variants (P5^3655^, P5^KR271^, P5^KR317^ and P5^P652^) was incubated with vitronectin (VN) alone or in the presence of C4BP and FH (*A, D*)*,* C4BP alone or in the presence of vitronectin and FH (*B, E*); and FH only, or in the presence of C4BP and vitronectin (*C, D*). All complement regulators were analysed at equal molarity, 25 nM, as a single or in a mixture of ligand. Complement regulators-binding assay was analysed by flow cytometry using CytoFlex flow cytometer (Beckman Coulter, Brea, CA) and the FlowJo software (Becton Dickison, Ashland, OR). Vitronectin, C4BP and FH bound on the bacteria surface was detected with mouse anti-human vitronectin, anti-human C4BP, and anti-human FH monoclonal antibodies (BioRad, Hercules, CA), respectively; and goat anti-mouse IgG-FITC (BioRad) as secondary antibody. Binding to complement regulators was measured as median fluorescence intensity (MFI) (*A-C*); or as percentage of binding population relative control condition without ligand (*D-F*). Naive *Escherichia coli* not expressing P5 was used as a negative control. Binding of P5 to vitronectin, C4BP or FH was not competed or inhibited by the presence of C4BP and FH, vitronectin and FH, and vitronectin and FH, respectively. This indicates, P5 variants can simultaneously bind vitronectin, C4BP and FH without interference by other complement regulators, except for P5^3655^-FH interaction. Statistically significant differences between bacteria incubated with single and multiple or without complement regulators were calculated by two-way ANOVA. *, *p*<0.05; **, *p*<0.01; ***, *p*<0.005; and ****, *p*<0.001.

| TABLE S1. List of primers used in current study | | |
| --- | --- | --- |
| Primer | **Sequence (5’-3’)** | **Amplicon** |
| Su317-F | TAAAGCTGATTGATTGATATTCAGGTAAATTTGCC | PCR fragment corresponding to the upstream flanking (UF) and downstream flanking regions of *ompP5* from KR317 that were fused to *cat.* The UF-*cat-*DF fragment was used for transformation into NTHi KR317 to yield KR317Δ*ompP5*. |
| Su317-R | ATGCCAAATCATTTACGTATAAGTATTGGTTTACC |  |
| SuP5_F^a^ | GTGCCCCATGGGCAAAAAAACTGCAATCGCATTAGTAGT | Open reading frame of P5 from NTHi KR317 and P652 for subsequent cloning into pET16b. Constructs were to be used for protein expression on the surface of *E. coli* BL21(DE). |
| SuP5_R^b^ | GTCTCCATATGTTATTTAGTACCGTTTACTGCGATTTC |  |

^a, b^ Restriction enzyme cutting sites are underlined. ­^a^*Nco*I, ^b^*Nde*I.

| **TABLE S2. Peptides for loop 1, loop 2, loop 3 and loop 4 of P5 variants** | | |  |
| --- | --- | --- | --- |
| **Peptide name^a^** | **Sequence** | **Number of residues** | **pI^b^** |
| **P5^NTHi3655^** |  |  |  |
| Loop 1 | HDGINNNGAIKEALTSASYGYRRN | 24 | 8.5 |
| Loop 2 | GRAKLREVGKPSAKHTNHG | 19 | 11.10 |
| Loop 3 | SDYKRYEEANGTRNHDKGRHSLRTS | 25 | 9.52 |
| Loop 4 | TRVGKLRTQDKPNSAINYNPWIG | 23 | 10.82 |
| **P5^KR271^** |  |  |  |
| Loop 1 | HDGINNNGVIGEALQSSGYGYRRN | 24 | 6.75 |
| Loop 2 | GRAKIREAGKPKAKHTNHG | 19 | 11.17 |
| Loop 3 | SDYKFYEVANGARDRNQGRHSLRTS | 25 | 9.69 |
| Loop 4 | TRVGKYRTQDKPNSAINYNPWIG | 23 | 9.99 |
| **P5^KR317^** |  |  |  |
| Loop 1 | HDGINNNGAIGEVLQSSGYGYRRN | 24 | 6.75 |
| Loop 2 | GRAKLREVGQTRAKHTNHG | 19 | 11.72 |
| Loop 3 | SDYKRYEEANGTRNHKEGRHSLRTS | 25 | 9.52 |
| Loop 4 | TRVGKFRTQDKPNSAINYNPWIG | 23 | 10.28 |
| **P5 ^P652^** |  |  |  |
| Loop 1 | HDGINNNGAIKKDLLGGFASYGYRR | 26 | 9.52 |
| Loop 2 | GRVKFRLAGKPKAKHTNHG | 19 | 12.03 |
| Loop 3 | SDYKFYEDANGTRNHKEGRHTARAS | 25 | 8.20 |
| Loop 4 | TRVGKYRPQDNPNTAINYNPWIG | 23 | 9.69 |
| **Mutated peptides** |  |  |  |
| **P5^3655^** |  |  |  |
| Loop 2^L5A-V8A-S12A^ | GRAKAREAGKPAAKHTNHG | 19 | 11.10 |
| **P5^KR271^** |  |  |  |
| Loop 2^I5A^ | GRAKAREAGKPKAKHTNHG | 19 | 11.17 |
| **P5^KR317^** |  |  |  |
| Loop 2^L5A-V8A-Q10A-T11A-R12A^ | GRAKAREAGAAAAKHTNHG | 19 | 11.00 |
| **P5^P652^** |  |  |  |
| Loop 2^V3A-F5A-L7A^ | GRAKARAAGKPKAKHTNHG | 19 | 12.03 |
| **Mutated at all non-conserved position** | |  |  |
| Loop 2^all-mutant^ | GRAKARAAGAAAAKHTNHG | 19 | 12.02 |

^a^ Peptide sequence of loop 1-4 were predicted based on Webb and Cripps [2].

^b^ Isoelectric point (pI) was determined with <https://web.expasy.org/compute_pi/>

**REFERENCES**

1. Su YC, Kadari M, Straw ML, et al. Non-typeable *Haemophilus influenzae* major outer membrane protein P5 contributes to bacterial membrane stability, and affects the membrane protein composition crucial for interactions with the human host. Front Cell Infect Microbiol **2023**; 13:1085908.

2. Webb DC, Cripps AW. Secondary structure and molecular analysis of interstrain variability in the P5 outer-membrane protein of non-typable *Haemophilus influenzae* isolated from diverse anatomical sites. J Med Microbiol **1998**; 47:1059-67.
